# Systematic review and assessment of validated case definitions for depression in administrative data

**DOI:** 10.1186/s12888-014-0289-5

**Published:** 2014-10-17

**Authors:** Kirsten M Fiest, Nathalie Jette, Hude Quan, Christine St. Germaine-Smith, Amy Metcalfe, Scott B Patten, Cynthia A Beck

**Affiliations:** Department of Community Health Sciences & Institute for Public Health, University of Calgary, 3280 Hospital Drive NW, Calgary, Alberta T2N4Z6 Canada; Department of Clinical Neurosciences & Hotchkiss Brain Institute, University of Calgary, 1403 29 Street NW, Calgary, AB T2N2T9 Canada; Department of Psychiatry & Mathison Centre for Mental Health Research and Education, University of Calgary, 4th Floor TRW Building, 3280 Hospital Drive NW, Calgary, AB T2N4Z6 Canada; Department of Obstetrics and Gynaecology, University of British Columbia, Vancouver, British Columbia Canada

**Keywords:** Depression, Validation, Systematic review, Administrative data, International Classification of Diseases

## Abstract

**Background:**

Administrative data are increasingly used to conduct research on depression and inform health services and health policy. Depression surveillance using administrative data is an alternative to surveys, which can be more resource-intensive. The objectives of this study were to: (1) systematically review the literature on validated case definitions to identify depression using International Classification of Disease and Related Health Problems (ICD) codes in administrative data and (2) identify individuals with and without depression in administrative data and develop an enhanced case definition to identify persons with depression in ICD-coded hospital data.

**Methods:**

(1) Systematic review: We identified validation studies using ICD codes to indicate depression in administrative data up to January 2013. (2) Validation: All depression case definitions from the literature and an additional three ICD-9-CM and three ICD-10 enhanced definitions were tested in an inpatient database. The diagnostic accuracy of all case definitions was calculated [sensitivity (Se), specificity (Sp), positive predictive value (PPV) and negative predictive value (NPV)].

**Results:**

(1) Systematic review: Of 2,014 abstracts identified, 36 underwent full-text review and three met eligibility criteria. These depression studies used ICD-9 and ICD-10 case definitions. (2) Validation: 4,008 randomly selected medical charts were reviewed to assess the performance of new and previously published depression-related ICD case definitions. All newly tested case definitions resulted in Sp >99%, PPV >89% and NPV >91%. Sensitivities were low (28-35%), but higher than for case definitions identified in the literature (1.1-29.6%).

**Conclusions:**

Validating ICD-coded data for depression is important due to variation in coding practices across jurisdictions. The most suitable case definitions for detecting depression in administrative data vary depending on the context. For surveillance purposes, the most inclusive ICD-9 & ICD-10 case definitions resulted in PPVs of 89.7% and 89.5%, respectively. In cases where diagnostic certainty is required, the least inclusive ICD-9 and −10 case definitions are recommended, resulting in PPVs of 92.0% and 91.1%. All proposed case definitions resulted in suboptimal levels of sensitivity (ranging from 28.9%-35.6%). The addition of outpatient data (such as pharmacy records) for depression surveillance is recommended and should result in improved measures of validity.

**Electronic supplementary material:**

The online version of this article (doi:10.1186/s12888-014-0289-5) contains supplementary material, which is available to authorized users.

## Background

Globally, depression is the second leading cause of life years spent with disability, and the third leading cause of disability-adjusted life-years [1,2]. Major depression is associated with decreased quality of life, productivity loss and high costs to the patient and society [3,4]. The 12-month prevalence of depression is 4.7% [5], with a lifetime prevalence of up to 16.6% in adults over 18 years [6].

The epidemiology of depression is traditionally studied using population-based surveys; however, these can only be conducted infrequently while also covering large geographic areas due to their labor intense and time consuming nature. Researchers are seeking alternative data sources for surveillance and population-based studies. Administrative health data, such as physician billing or hospital discharge data, are increasingly being used for epidemiologic and health services research [7-9]. These data are typically coded using the World Health Organization (WHO) International Classification of Diseases and Related Health Problems (ICD) codes, with the ICD-9 and ICD-10 classifications being the most widely used international coding systems [9,10]. There is concern that misclassification of diagnosis in these data can potentially introduce bias into results obtained from their analysis. In spite of this, there is no widely used or agreed upon validated case definition of depression employed by researchers who use administrative data.

The aims of the current study were to: (1) identify validated ICD-9 & 10 depression case definitions in administrative data through a systematic review of the literature; (2) identify individuals with and without depression in administrative data and develop an enhanced case definition to identify persons with depression in ICD-coded hospital data and; (3) assess the diagnostic accuracy of these case definitions compared to a reference standard.

## Methods

### Study 1. Systematic review of validated depression case definitions

#### Search strategy

We searched Medline (1948 to January 2013) and Embase (1980 to January 2013) for relevant articles. Our search strategy consisted of the following sets of terms: (1) [administrative data or hospital discharge data or ICD-9 or ICD-10 or medical record or health information or surveillance or physician claims or claims or hospital discharge or coding or codes] AND (2) [validity or validation or case definition or algorithm or agreement or accuracy or sensitivity or specificity or positive predictive value or negative predictive value] AND (3) the medical subject heading terms for depression. Searches were limited to human studies published in English. The broad nature of the search strategy allowed for the detection of modifications of ICD codes, such as international clinical modification (e.g. ICD-9-CM).

#### Study selection

Two reviewers (CB and AM) independently screened all titles and abstracts to identify those meeting pre-determined eligibility criteria. To be considered, articles had to report on original research that validated specified ICD-9 or ICD-10 coding for depression against a reference standard, and had to report at least one of the following estimates of validity: sensitivity, specificity, positive predictive value (PPV) or negative predictive value (NPV). Articles that validated depression in specialized populations (e.g. diabetes or multiple sclerosis) were excluded to ensure the case definitions would be more generalizable to the general population. Papers that did not employ solely medical encounter data in their definitions (eg. the inclusion of pharmacy data) were also excluded as such data are often unavailable to researchers or do not use ICD-coded data.

Full-text article review was performed by two reviewers (AM or CSS, and CB) for all abstracts identified as eligible for inclusion by either reviewer in the abstract screening stage. Disagreements of eligibility were resolved by a third reviewer (KF or NJ). Bibliographies of included articles were manually searched for additional articles which were then screened and reviewed using the same methods. Local administrative data and depression experts were contacted and asked to identify any additional missing studies.

#### Data extraction

Two reviewers (CSS and KF) independently extracted and reached agreement on data from the included articles using a standardized data abstraction form. Disagreements were resolved by a third reviewer when necessary (NJ). Validated ICD-9 or ICD-10 based case definitions were abstracted from each paper, along with estimates of sensitivity, specificity, PPV and NPV. The following data were also extracted: study information (author, year, country), population demographics (population size, time of data collection), prevalence of depression, type of administrative database, and a description of the reference standard used to validate the depression case definition.

#### Quality assessment

Two reviewers (CSS and KF) assessed study quality using a standardized 40-item checklist [11] designed to assess the quality of validation studies of administrative data (Additional file 1). One point was assigned for fulfilling each item on the checklist. If it was unclear whether an item was fulfilled, it was marked as uncertain and no points were given. Questions on the scale relate to methodology (validation cohort description, sampling procedures, study flow diagram) and the reporting of results (based on disease severity, likelihood ratios, subgroup analyses, 95% confidence intervals). Some items on the checklist were not applicable due to the nature of the study. Disagreements were resolved by consensus.

### Study 2. Validation of depression case definitions in hospital discharge data

#### Administrative data source

We had access to data from the discharge abstract database (DAD), a hospitalization database used in all adult hospitals in Calgary, Alberta, Canada, to test the previously published and new case definitions for depression. The DAD includes data on patient demographics, procedures, and principal diagnosis and comorbidities (whether pre-existing and new). Up to 50 diagnoses are coded per hospitalization, including one primary/main diagnosis; only 25 diagnoses are available for release to researchers although it is extremely rare for a patient to have more than 25 diagnoses coded in their DAD record. All records for those patients aged 18 years and over admitted between January 1, 2003 and June 30, 2003 were obtained from this ICD-10 coded data. For patients with multiple admissions in that period, only the first (index) admission of the six-month period was used in the analyses. Ethical approval was granted by the University of Calgary Conjoint Health Research Ethics Board.

#### Study population and chart review

We randomly selected 4,008 inpatient records from the DAD for patients who were admitted for any indication between January 1 and June 30, 2003 [12]. As no psychiatric hospitals exist in Calgary, this sample can be assumed to include a representative series of admissions for psychiatric and non-psychiatric indications. Charts for each of these 4,008 index visits were subsequently reviewed and diagnoses extracted by two trained chart reviewers with nursing backgrounds. The entire chart was examined in duplicate, including the cover page, discharge summaries, admission notes, consultation reports, physician orders and diagnostic reports. The entire review process took approximately one hour per chart to complete. The presence or absence of depression was based on all documented information in the chart. Detailed methodology describing the study population and chart review has been published elsewhere [12].

#### Coding algorithms

A total of 11 ICD-based case definitions (algorithms) of depression were tested in the DAD: five algorithms from the studies identified through systematic review, and six new, more inclusive, algorithms developed by the study investigators (neurologist and psychiatrists) (Table 1). The new algorithms included three ICD-9-CM case definitions with increasing levels of ICD-9-CM code inclusivity. Algorithm 1 for ICD-9-CM was the least inclusive: 296.20-296.25 (Major Depressive Disorder, single episode: unspecified, mild, moderate, severe without psychotic behavior, severe with psychotic behavior, in partial remission), 296.30-296.35 (Major Depressive Disorder, recurrent episode: unspecified, mild, moderate, severe without psychotic behavior, severe with psychotic behavior, in partial remission), 300.4 (Dysthymic Disorder) & 311 (Depressive Disorder not Elsewhere Classified); algorithm 2 was moderately inclusive: algorithm 1 plus 296.5 (Bipolar I Disorder, most recent episode depressed), 296.6 (Bipolar I Disorder, most recent episode mixed), 296.82 (Atypical Depressive Disorder) & 296.90 (Unspecified Episodic Mood Disorder); algorithm 3 was the most inclusive: algorithm 2 plus 309.0 (Adjustment Disorder with depressed mood), 309.1 (Prolonged Depressive Reaction) & 309.28 (Adjustment Disorder with mixed anxiety and depressed mood).

**Table 1:** **Validation of International Classification of Disease (ICD) hospital discharge abstract data based on chart review data for depression**

**Case definition**	**Codes employed**	**Sensitivity (%) (95% CI)**	**Specificity (%) (95% CI)**	**Positive predictive value (%) (95% CI)**	**Negative predictive value (%) (95% CI)**
Alaghehbandan (2012) 1	ICD-9: 311	19.92 (16.42-23.79)	99.69 (99.44-99.84)	89.62 (82.19-94.70)	90.21 (89.23-91.13)
Alaghehbandan (2012) 2	ICD-10-CA: F32.0-F32.3, F32.8, F32.9, F33.0-F33.4, F33.8, F34.1	29.56 (25.50-33.88)	99.58 (99.30-99.76)	90.38 (84.64-94.52)	91.28 (90.34-92.15)
Noyes (2011) 1	ICD-9: 296.20-.24, 296.30.-34	1.05 (0.34-2.43)	100.00 (99.90-100.00)	100.00 (47.82-100.00)	88.21 (87.17-89.19)
Noyes (2011) 2	ICD-9: 296.20-.24, 296.30-.34, 300.4, 311	28.93 (24.90-33.23)	99.66 (99.41-99.82)	92.00 (86.44-95.80)	91.21 (90.28-92.09)
Singh (2006)	ICD-9: 296.xx, 300.4, 301.1x, 298, 311	28.90 (24.9-33.2)	99.70 (99.40-99.80)	92.00 (86.40-95.80)	91.20 (90.30-92.10)
ICD-9-CM (new definitions tested)					
Case Definition 1*	296.20-.25, 296.30-.35, 300.4, & 311	28.93 (24.90-33.23)	99.66 (99.41-99.82)	92.00 (86.44-95.80)	91.21 (90.28-92.09)
Case Definition 2*	CD1 + 296.5, 296.6, 296.82 & 296.90	29.14 (25.10-33.34)	99.52 (99.23-99.72)	89.10 (83.13-93.52)	91.23 (90.29-92.10)
Case Definition 3*	CD2 + 309.0, 309.1 & 309.28	32.91 (28.71-37.33)	99.49 (99.20-99.70)	89.71 (84.23-93.79)	91.65 (90.73-92.51)
ICD-10 (new definitions tested)					
Case Definition 4**	F32.0-32.9, F33.0-33.3, F33.8, F33.9, F34.1 & F41.2	34.17 (29.92-38.62)	99.55 (99.27-99.74)	91.06 (85.89-94.80)	91.80 (90.89-92.65)
Case Definition 5**	CD4 + F31.3-F31.6	34.59 (30.32-39.05)	99.52 (99.23-99.72)	90.66 (85.47-94.46)	91.85 (90.93-92.69)
Case Definition 6**	CD5 + F34.8, F34.9, F38.0, F38.1, F38.8, F39, F99	35.64 (31.34-40.12)	99.43 (99.13-99.65)	89.47 (84.21-93.45)	91.96 (91.05-92.80)

CD: Case Definition, ICD: International Classification of Disease.

*CD 1: most restrictive, CD 2: moderately inclusive, CD 3: most inclusive.

**CD 4: most restrictive, CD 5: moderately inclusive, CD 6: most inclusive.

Similarly, three new ICD-10-CA based algorithms were developed, also with differing levels of inclusivity. Algorithm 1 for ICD-10-CA was the least inclusive: F32.0-32.9 (Depressive Episode: mild, moderate, severe without psychotic symptoms, severe with psychotic symptoms, other depressive episode, unspecified), F33.0-33.3 (Recurrent Depressive Episode: mild, moderate, severe without psychotic symptoms, severe with psychotic symptoms), F33.8 (Other Recurrent Depressive Disorder), F33.9 (Recurrent Depressive Disorder, Unspecified), F34.1 (Dysthymia) & F41.2 (Mixed Anxiety and Depressive Disorder); algorithm 2 was moderately inclusive: algorithm 1 plus F31.3-31.6 (Bipolar Affective Disorder: mild or moderate depression, severe depression without psychotic symptoms, severe depression with psychotic symptoms, mixed episode); algorithm 3 was most inclusive: algorithm 2 plus F34.8 (Other Persistent Mood Disorders), F34.9 (Persistent Mood Disorder, unspecified), F38.0 (Other Single Mood Disorders), F38.1 (Other recurrent Mood Disorders), F38.8 (Other Specified Mood Disorders), F39 (Unspecified Mood Disorders) & F99 (Mental Disorder, not elsewhere classified). For all algorithms, the presence of only one of the above diagnostic codes in any of the diagnostic positions in the DAD was defined as sufficient to meet the case definition for depression.

We developed a map of depression related ICD codes (Table 2) between ICD-9-CM and ICD-10-CA to be used in our algorithms to enhance comparability and to guide the usage of both coding systems. Two psychiatrists (CB, SBP) and an expert in administrative data and neurologist (NJ) determined the mapping between ICD-9-CM and ICD-10-CA by reviewing clinical definitions and coding descriptions.

**Table 2:** **Included International Classification of Disease (ICD) codes and roadmap of ICD-10-CA to ICD-9-CM**

**ICD-10-CA Code**	**Definition**		**ICD-9-CM Code**	**Definition**
F31.31	Bipolar affective disorder, current episode mild or moderate depression	<=>	296.51	Bipolar I disorder, most recent episode (or current) mild depression
F31.32	Bipolar affective disorder, current episode mild or moderate depression	<=>	296.52	Bipolar I disorder, most recent episode (or current) moderate depression
F31.4	Bipolar affective disorder, current episode severe depression without psychotic symptoms	<=>	296.53	Bipolar I disorder, most recent episode (or current) severe without mention of psychotic behavior
F31.5	Bipolar affective disorder, current episode severe depression with psychotic symptoms	<=>	296.54	Bipolar I disorder, most recent episode (or current) severe specified as with psychotic behavior
F31.6x	Bipolar affective disorder, current episode mixed	<=>	296.6x	Bipolar I disorder, most recent episode (or current) mixed
F31.9	Bipolar affective disorder, current episode unspecified			
F32.0	Mild depressive episode	<=>	296.21	Major depressive disorder, single episode- mild
F32.1	Moderate depressive episode	<=>	296.22	Major depressive disorder, single episode- moderate
F32.2	Severe depressive episode without psychotic symptoms	<=>	296.23	Major depressive disorder, single episode- severe, without mention of psychotic behavior
F32.3	Severe depressive episode with psychotic symptoms	<=>	296.24	Major depressive disorder, single episode- severe, specified as with psychotic behavior
F32.4	Depressive disorder, single episode, in partial remission	<=>	296.25	Major depressive disorder, single episode- in partial or unspecified remission
F32.5	Depressive disorder, single episode, in full remission	<=>	296.26	Major depressive disorder, single episode- in full remission
F32.8	Other depressive episodes	<=>	296.82	Atypical depressive disorder
F32.9	Depressive episode, unspecified	<=>	296.20	Major depressive disorder, single episode- unspecified
F32.9	Depressive episode, unspecified	<=>	311	Depressive disorder, not elsewhere classified
F33.0	Recurrent depressive disorder, current episode mild	<=>	296.31	Major depressive disorder, recurrent episode- mild
F33.1	Recurrent depressive disorder, current episode moderate	<=>	296.32	Major depressive disorder, recurrent episode- moderate
F33.2	Recurrent depressive disorder, current episode severe without psychotic symptoms	<=>	296.33	Major depressive disorder, recurrent episode- severe, without mention of psychotic behavior
F33.3	Recurrent depressive disorder, current episode severe with psychotic symptoms	<=>	296.34	Major depressive disorder, recurrent episode- severe, specified as with psychotic behavior
F33.41	Recurrent depressive disorder, currently in remission	<=>	296.35	Major depressive disorder, recurrent episode- in partial or unspecified remission
F33.42	Recurrent depressive disorder, currently in remission	<=>	296.36	Major depressive disorder, recurrent episode- in full remission
F33.8	Recurrent depressive disorder, other			
F33.9	Recurrent depressive disorder, unspecified	<=>	296.30	Major depressive disorder, recurrent episode- unspecified
F34.1	Dysthymia	<=>	300.4	Dysthymic disorder
F34.8	Other persistent mood disorders	<=>		
F34.9	Persistent mood disorder, unspecified	<=>		
F38.0	Other single mood disorders	<=>		
F38.1	Other recurrent mood disorders	<=>		
F38.8	Other specified mood disorders	<=>	296.99	Other specified episodic mood disorder
F39	Unspecified mood disorder	<=>	296.90	Unspecified episodic mood disorder
F41.2	Mixed anxiety and depressive disorder	<=>		
F43.2	Adjustment Disorders	<=>	309.0	Adjustment disorder with depressed mood
F43.2	Adjustment Disorders	<=>	309.28	Adjustment disorder with mixed anxiety and depressed mood
F99	Mental disorder, not elsewhere specified			
			296.82	Atypical depressive disorder
			298	Other non-organic depressive psychoses
			309.1	Prolonged depressive reaction
NOTE: ICD = International Classification of Disease				

#### Statistical analyses

Estimates of sensitivity, specificity, PPV and NPV were calculated for all of the algorithms listed in Table 1. Sensitivity was defined as the proportion of people who had a depression diagnosis detected in the chart review who also had an ICD code for depression in the DAD. Specificity was defined as the proportion of people who did not have a depression diagnosis coded in the chart and who also did not have an ICD code for depression in the DAD. PPV was defined as the proportion of people with an ICD code for depression who also had a diagnosis of depression in the chart. NPV was defined as the proportion of people without an ICD code for depression who did not have a diagnosis of depression in the chart. All data analyses were conducted in STATA version 11.1 [13].

## Results

### Study 1. Systematic review of validated depression case definitions

#### Identification and description of studies

**Figure 1:**
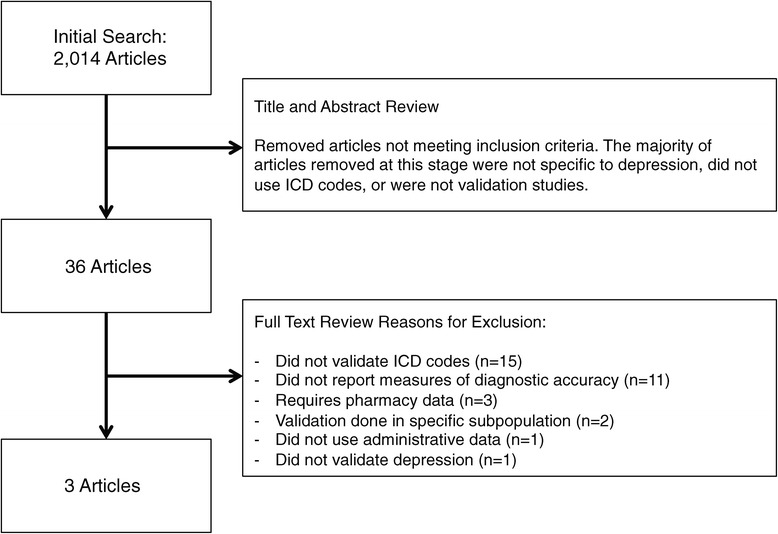
**Study flow diagram.**

A total of 2,014 abstracts were identified and 36 articles were reviewed in full text, of which three articles met all eligibility criteria (Figure 1, Additional file 2). Only one study included ICD-10 codes [14]. The reference standards used to validate the depression diagnosis included electronic medical records, medical chart review, a clinical interview and depression questionnaire, and self-report mail questionnaire (including questions on a physician diagnosis of a chronic condition, demographic information, smoking status, functional limitations and the veterans version of the Short Form-36 (SF-36)). Characteristics of the three included studies can be found in Table 3.

**Table 3:** **Validation studies and results of articles from the systematic review**

**Author**	**Country**	**N**	**Year of data collection**	**Administrative database**	**Reference standard**	**ICD codes**	**Prevalence (%)**	**Sensitivity (%)**	**Specificity (%)**	**PPV (%)**	**NPV (%)**
Alaghehbandan (2012)	Canada	510	1995-2008	Provincial Hospital Separation Database; Provincial fee-for-service physician claims database	Electronic Medical Record and Medical Chart Review	ICD-9: 311; ICD-10-CA: F32.0-F32.3, F32.8, F32.9, F33.0-F33.4, F33.8, F34.1	NR				
						≥ 1 Hospital OR ≥2 Physician within 1 Year		70.0	93.8	91.7	76.0
						≥ 1 Hospital OR ≥2 Physician within 2 Years		75.1	93.0	91.3	79.1
						( ≥1 Hospital OR ≥1 PSY) OR ≥2GP within 1 Year		73.1	93.4	91.6	77.9
						( ≥1 Hospital OR ≥1 PSY) OR ≥2 GP within 2 Years		77.5	93.0	91.6	80.7
						( ≥1 Hospital OR ≥1 PSY) OR ≥3 GP within 3 Years		69.6	93.4	91.2	75.7
Noyes (2011)	USA	1,605	1998-2002	Medicare	Previous MDE, GDS, or MINI-MDE	ICD-9: 296.20-.24, 296.30-.34	18% based on MINI				
					MDE at baseline- claims diagnoses at 6 months before & after baseline			14.13	95.61	41.05	83.72
					MDE at baseline- claims diagnoses at 12 months before & after baseline			18.12	93.45	37.31	84.14
					GDS ≥6 at baseline- claims diagnoses at 6 months before & after baseline			9.96	95.60	55.42	65.90
					GDS ≥6 at baseline- claims diagnoses at 12 months before & after baseline			12.69	93.26	50.86	66.03
					MINI-MDE at baseline- claims diagnoses at 6 months before and after baseline	ICD-9: 296.20-.24, 296.30-.34, 300.4, 309.0, 311		33.70	82.12	28.97	85.12
					MINI-MDE at baseline- claims diagnoses at 12 months before and after baseline			46.38	74.98	28.51	86.67
					GDS ≥6 at baseline- claims diagnoses at 6 months before and after baseline			27.92	83.35	47.96	67.79
					GDS ≥6 at baseline- claims diagnoses at 12 months before and after baseline			39.57	77.42	49.07	69.98
					GDS ≥11 at baseline- claims diagnoses at 6 months before and after baseline	ICD-9: 296.20-.24, 296.30-.34		55.06	73.32	13.07	95.73
					GDS ≥11 at baseline- claims diagnoses at 12 months before and after baseline			42.48	78.57	40.37	80.00
					2 positive MINI-MDEs at baseline and at 12 months- claims diagnoses at 12 months before and after baseline			46.63	74.69	12.11	94.89
Singh (2006)	USA	40,508	1996-1998	Veterans Integrated Service Network	Self-report of depression	ICD-9: 296.xx, 300.4, 301.1x, 298, 311	NR	34.00	97.00	83.00	79.00

PPV: Positive predictive value; NPV: Negative predictive value; MDE: Major Depressive Episode; MINI-MDE: Mini-International Neuropsychiatric Interview-Major Depressive Episode; GDS: Geriatric Depression Scale; NR: Not reported; PSY: Psychiatrist visit; GP: General Practitioner visit.

The reference standard used to validate the depression diagnosis differed between the studies: Alaghehbandan [14] used electronic medical records and medical chart review, Noyes [15] used a clinical interview and depression questionnaire, while Singh [16] used a self-report mail questionnaire. Sensitivity, specificity, PPV and NPV were reported in all papers: sensitivities ranged from 9.9% to 55.1% [15], 34.0% [16] and 69.6% to 77.5% [14]; specificities ranged from 73.3% to 95.6% in one paper [15], while the Singh [16] paper reported a specificity of 97.0% and the Alaghehbandan [14] paper specificities ranging from 93.0 to 93.8%; PPVs ranged from 12.1% to 55.4% [15], 83.0% [16] and 91.2% to 91.7% [14], while NPVs ranged from 65.9% to 95.7% [15], 75.7% to 80.7% [14] and 79.0% [16]. The prevalence of depression was reported in one study, at 18% [15]. Meta-analysis was not considered due to substantial heterogeneity in the administrative databases and differing reference standards employed to validate the depression diagnoses.

#### Study quality assessment

Based on Benchimol’s quality rating scale [11], the Noyes [15] study had the highest quality score (25/41), followed by Singh [16] with 24 out of 41 points and Alaghehbandan [14] with 22 out of 41 points (Additional file 1). All of the articles identified themselves as a study reporting on and including the following: (1) diagnostic accuracy and administrative data; (2) disease identification and validation; and (3) a description of the validation cohort. Every article described the methods of calculating diagnostic accuracy and reported at least four estimates of diagnostic accuracy. Each article also discussed the applicability of their findings to the general population. Points were lost because studies did not provide detailed information on the methodology used in the validation (eg. Inclusion/exclusion criteria, recruitment procedures).

### Study 2. Validation of depression case definitions in hospital discharge data

Four hundred and seventy-seven of the 4,008 inpatient charts reviewed indicated the presence of depression, resulting in a hospital-based prevalence of 11.9% (95% Confidence Interval (CI): 10.9-12.9). The five previously validated case definitions [14-16] (Table 1), when applied to the DAD data, resulted in specificity estimates above 99.5%, PPVs of almost 90% and higher, and NPVs greater than 88%. Sensitivities ranged from 1.1% - 28.9%.

Three new case definitions for ICD-9-CM and three for ICD-10-CA codes were examined using DAD data (Table 1). For ICD-9, all newly developed case definitions resulted in specificities of over 99%, PPVs of at least 89% and NPVs of over 91%. Sensitivities ranged from 28.9-32.9%, with the greatest sensitivity resulting from the most inclusive case definition (#3). Measures of validity were similar for ICD-10 coding: specificities were greater than 99%, PPVs were at least 89% and NPVs were well over 91%. Sensitivities were slightly better for the three ICD-10 based case definitions, relative to ICD-9, ranging from 34.2-35.6%, with the greatest sensitivity resulting from the most inclusive case definition (#6).

Thirty-two (6.7%) of the 477 depressed persons identified by chart review had a primary diagnosis of depression at the index visit; the remaining 445 depressed persons had ICD codes for depression in secondary diagnostic positions. False negative cases, rather than being coded by one of the depression-related ICD-9-CM or ICD-10 codes in Table 2, were all found to be coded in the DAD as follows: 290.x (Dementias), 300.00 (Neuroses), 301.x (Personality Disorders), 303.x (Alcohol Dependence) or 304.x (Drug Dependence).

## Discussion

Researchers often use a variety of administrative data sources to identify cases to answer important health research questions. The most commonly used data sources include hospital discharge data and physician claims data. This study was part of a larger study validating case definitions for a number of conditions in hospital data. Through a systematic review of the literature, two validated ICD-9 based case definitions and one combined ICD-9/ICD-10-CA validation were identified and the validity of the definitions was further investigated in a new dataset. We tested an additional six new case definitions to determine the best algorithm for identifying depression in our administrative hospitalization data. Although physician (including outpatient visits) data were not validated as part of this study, the findings are still important. We demonstrate that if one was interested in studying health outcomes in those admitted with a diagnosis of depression based on administrative data, there is a strong chance that they will have depression. The choice of depression case definitions depends on the purpose for which the data will be used.

For surveillance purposes, sensitivity and PPV need to be balanced so that they are both adequately high. In surveillance, we suggest employing case definition #3 (all definitions are defined in Table 1) for ICD-9 codes and case definition #6 for ICD-10 codes, based on our results. These two algorithms maximize sensitivity without an accompanying loss of specificity. The sensitivity however, is still suboptimal for surveillance purposes in inpatient data. In situations where diagnostic certainty is necessary (eg. The selection of cases in a case–control study, where finding every case is not essential, but ensuring all cases have the disease is), we would recommend case definition #1 for ICD-9-CM (similar to the definition Noyes [15]) and case definition #4 for ICD-10. The Noyes [15] algorithm is similar to our recommended case definition #1, except it does not assess cases currently in remission; the purpose of the new definition was to identify all cases of depression. Specificities were highest for these two new algorithms (#1 and #4), improving the likelihood that those who are not depressed will be identified correctly. All newly tested algorithms for both ICD-9-CM and ICD-10 had high specificities. High specificities are encouraging; in medical coding, there is a risk that unrelated terms containing the word “depression”, such as respiratory depression or QT interval depression could be miscoded; our results support that this did not occur. As depression is an episodic and recurrent condition, many individuals with well-established diagnoses may be asymptomatic at the time of presentation for another condition (as evidenced by the 93% of depressed persons having their diagnosis in a secondary position). Administrative data may be better at detecting active cases of depression rather than all individuals with a history of depression.

In 2012 Townsend and colleagues published a systematic review of validated methods for identifying depression using administrative data [17]. The search of the Townsend review was conducted in the summer of 2010; since then additional eligible papers have been published. The present study reviewed multiple databases and includes articles published until January 2013. Only papers validating ICD-9 codes were included in the Townsend review, and the authors further restricted inclusion by only allowing the codes 296.2 (major depressive disorder- single episode), 296.3 (major depressive disorder- recurrent episode), 300.4 (dysthymic disorder), 311 (depressive disorder not elsewhere classified), 298.0 (depressive type psychosis) and 309.1(prolonged depressive reaction). The current review validated combinations of 19 ICD-9-CM codes and 27 ICD-10 codes. Differing inclusion/exclusion criteria (i.e. the use of prescription drug data in the case definition and validation of coding in specialized subpopulations such as diabetes) between Townsend’s review and the current study probably explain the discrepancy in the number of included full-text articles (10 versus 3, respectively). The Townsend review reported that most algorithms resulted in only slight to fair levels of agreement with the reference standard. They hypothesize that the inadequate performance of these scales may in part be due to a lack of clinical detection of depression in hospitals.

The prevalence of depression in our hospital-based population (11.9%) is consistent with previous inpatient literature [18,19], though higher than most studies of individuals with medical conditions in the general population [20]. Patients in our sample rarely have depression coded in the primary diagnostic position, suggesting they presented to hospital for a different indication, and have a co-existing medical condition. This is not surprising as persons with chronic health conditions are more likely to have depression than their healthy counterparts [20,21]. This finding highlights the importance of examining all diagnostic positions when attempting to identify cases of depression in administrative data.

This analysis had several limitations. The literature review was limited to studies published in English and only in peer-reviewed journals (not the grey literature). It is thus possible the search did not identify all potential studies, as no formal assessment of publication bias was conducted. However, we did search two reference databases known to target articles with different characteristics (Medline and Embase). For the validation of administrative data case definitions, only discharge abstract (hospitalization) data were used, as physician claims data were not available; those patients who were most sick may have been selectively included, which may over-represent the prevalence of depression. On the other hand, a large number of charts (>4,000) were reviewed to decrease sampling variability in this setting. The validity of the coding may differ in other countries where coding is completed by physicians rather than by trained health technologists. However, our coders are highly skilled- in Canada, all professional coders undergo standardized national training [22]- which is a strength of this study. In spite of this, coders do depend on accurate and complete physician documentation in order to code each chart. As such, limited chart documentation could have resulted in undercoding; however, this is likely to occur in most jurisdictions. In addition, the results may not be generalizable to other jurisdictions where coding rules may differ- there may be fewer secondary diagnostic positions available for coding in other regions, and sensitivity may prove to be even lower in these locations. Though the health data used in this study is over 10 years old, there were no interventions or changes in the process of medical data registration in the study setting since that time. Finally, our study included only ICD codes and did not include other specialized data (such as pharmacy data). We specifically chose this approach to make the results more generalizable to other locations. In spite of these limitations, this study has numerous strengths. We improved upon existing case definitions by enhancing the sensitivity of depression case definitions. We also developed a map between ICD-9-CM and ICD-10 coding for the psychiatric codes tested in our algorithms.

## Conclusions

This systematic review and validation study examined new and existing case definitions for depression case ascertainment in administrative data. We suggest that validated case definitions be employed in all research involving depression in administrative data. Standardizing depression case definitions in administrative data is very important for study comparison; we recommend using and testing the ICD codes proposed here. Identifying depression in administrative data is challenging: it is an episodic condition that changes over time, so a different time span of observation may have a profound effect on results. In addition, the reference standards used for diagnosing depression have been highly variable and not as well defined as in some other chronic conditions. Case definitions should be selected based upon the objective of the study, as some algorithms maximize case finding and others enhance diagnostic certainty. To be adequate for surveillance purposes, researchers must consider multiple data sources to identify cases with depression, including for example inpatient (validated in this study) and outpatient claims data to optimize sensitivity. Thus, future studies validating physician claims data are warranted. Improvements to the coding of psychiatric conditions in ICD-11 will assist in the standardization of this approach. Future research should focus on further validating depression case definitions using additional data sources (eg. linkage with outpatient and pharmacy data, where available), with an eventual goal of increased sensitivity and specificity and consistent application of ICD codes for depression across disciplines.

## Additional files

Additional file 1:
**Quality Assessment of Included Articles from the Systematic Review.** This file presents a table of all included full-text articles and their ratings on each item of the study quality assessment. Additional file 2:
**Reasons for Systematic Review Full-Text Article Exclusion.** This file presents a table of all excluded full-text articles from the systematic review and the reasons for their exclusion.

## Abbreviations

DAD: Discharge abstract database
DSM: Diagnostic and statistical manual for mental disorders
CA: Canadian enhancement
CM: Clinical modification
ICD: International classification of diseases and related health problems
NPV: Negative predictive value
PPV: Positive predictive value
Se: Sensitivity
Sp: Specificity
WHO: World Health Organization

## References

[1] Murray CJ, Vos T, Lozano R, Naghavi M, Flaxman AD, Michaud C, Ezzati M, Shibuya K, Salomon JA, Abdalla S, Aboyans V, Abraham J, Ackerman I, Aggarwal R, Ahn SY, Ali MK, Alvarado M, Anderson HR, Anderson LM, Andrews KG, Atkinson C, Baddour LM, Bahalim AN, Barker-Collo S, Barrero LH, Bartels DH, Basanez MG, Baxter A, Bell ML, Benjamin EJ (2012). Disability-adjusted life years (DALYs) for 291 diseases and injuries in 21 regions, 1990–2010: a systematic analysis for the Global Burden of Disease Study 2010. Lancet.

[2] Vos T, Flaxman AD, Naghavi M, Lozano R, Michaud C, Ezzati M, Shibuya K, Salomon JA, Abdalla S, Aboyans V, Abraham J, Ackerman I, Aggarwal R, Ahn SY, Ali MK, Alvarado M, Anderson HR, Anderson LM, Andrews KG, Atkinson C, Baddour LM, Bahalim AN, Barker-Collo S, Barrero LH, Bartels DH, Basanez MG, Baxter A, Bell ML, Benjamin EJ (2012). Years lived with disability (YLDs) for 1160 sequelae of 289 diseases and injuries 1990–2010: a systematic analysis for the Global Burden of Disease Study 2010. Lancet.

[3] Greenberg PE, Kessler RC, Birnbaum HG, Leong SA, Lowe SW, Berglund PA, Corey-Lisle PK (2003). The economic burden of depression in the United States: How did it change between 1990 and 2000?. J Clin Psychiatry.

[4] Luppa M, Heinrich S, Angermeyer MC, Konig H, Riedel-Heller SG (2007). Cost-of-illness studies of depression: a systematic review. J Affect Disord.

[5] Pearson C, Janz T, Ali J: **Mental and substance use disorders in Canada.** *Health Glance* 2013, **Catalogue number 82-624-X**

[6] Kessler RC, Chiu WT, Demler O, Merikangas KR, Walters EE (2005). Prevalence, severity, and comorbidity of 12-month DSM-IV disorders in the National Comorbidity Survey Replication. Arch Gen Psychiatry.

[7] Kwon C, Liu M, Quan H, Thoo V, Wiebe S, Jette N (2011). Motor vehicle accidents, suicides, and assaults in epilepsy: a population-based study. Neurology.

[8] Patorno E, Bohn R, Wahl P, Avorn J, Partrick A, Liu J, Schneeweiss S (2010). Anticonvulsant medications and the risk of suicide, attempted suicide, or violent death. J Am Med Assoc.

[9] Jette N, Quan H, Hemmelgarn B, Drosler S, Maass C, Oec D, Moskal L, Paoin W, Sundararajan V, Gao S, Jakob R, Ustun B, Ghali WA (2010). The development, evolution, and modifications of ICD-10: challenges to the international comparability of morbidity data. Med Care.

[10] Organization WH (2012). World Health Statistics 2012.

[11] Benchimol EI, Manuel DG, To T, Griffith AM, Rabeneck L, Guttmann A (2011). Development and use of reporting guidelines for assessing the quality of validation studies of health administrative data. J Clin Epidemiol.

[12] Quan H, Li B, Saunders D, Parsons G, Nilsson C, Alibhai A, Ghali WA (2008). Assessing validity of ICD-9-CM and ICD-10 adminsitrative data in recording clinical conditions in a unique dually coded database. Health Serv Res.

[13] *STATA Corp 11.1.* College Station, TX: STATA Corp.; 2012.

[14] Alaghehbandan R, MacDonald D, Barrett B, Collins K, Chen Y (2012). Using administrative databases in the surveillance of depressive disorders- case definitions. Popul Health Manage.

[15] Noyes K, Liu H, Lyness JM, Friedman B (2011). Medicare beneficiaries with depression: comparing diagnoses in claims data with the results of screening. Psychiatr Serv.

[16] Singh JA (2009). Accuracy of veterans affairs databases for diagnoses of chronic disease. Prevelnting Chronic Disease: Public Health Research, Practice, and Policy.

[17] Townsend L, Walkup JT, Crystal S, Olfson M (2012). A systematic review of validated methods for identifying depression using adminstrative data. Pharmacoepidemiol Drug Saf.

[18] Rentsch D, Dumont P, Borgacci S, Carballeira Y, de Tonnac N, Archinard M, Andreoli A (2007). Prevalence and treatment of depression in a hospital department of internal medicine. Gen Hosp Psychiatry.

[19] Sharma P, Avasthi A, Chakrabarti S, Varma S (2002). Depression among hospitalised medically ill patients: a two-stage screening study. J Affect Disord.

[20] Patten SB, Beck CA, Kassam A, Williams JVA, Barbui C, Metz LM (2005). Long-term medical conditions and major depression: strength of association for specific conditions in the general population. Can J Psychiatr.

[21] Jette N, Patten S, Williams J, Becker W, Wiebe S (2008). Comorbidity of migraine and psychiatric disorders- a national population-based study. Headache.

[22] **Data and standards.** [http://www.cihi.ca/CIHI-ext-portal/internet/EN/SubTheme/standards+and+data+submission/standards/cihi010688]

