# Supplementary material for: Systematic review and assessment of validated case definitions for depression in administrative data
Source: BMC Psychiatry. 2014 Oct 17;14:289. doi: 10.1186/s12888-014-0289-5 (PMC4201696; doi:10.1186/s12888-014-0289-5)
Supplement: Additional file 1: — Quality Assessment of Included Articles from the Systematic Review. This file presents a table of all included full-text articles and their ratings on each item of the study quality assessment. [file 12888_2014_289_MOESM1_ESM.docx]

Additional File 1. Quality Assessment[11] of Included Articles from the Systematic Review

| Quality Item | Noyes 2011 | Alaghehbandan  2012 | Singh 2009 |
| --- | --- | --- | --- |
|  |  |  |  |
| 1. Identifies article as study of assessing diagnostic accuracy? | Yes | Yes | Yes |
| 2. Identifies article as study of administrative data? | Yes | Yes | Yes |
| 3. States disease identification and validation as one of the goals of study? | Yes | Yes | Yes |
| 4. Describes validation cohort? (cohort of patients to which reference standard was applied) | Yes | Yes | Yes |
| 4a. Age? | Yes | Yes | No |
| 4b. Disease? | Yes | Yes | Yes |
| 4c. Severity? | Yes | Yes | No |
| 4d. Location/ jurisdiction? | Yes | Yes | Yes |
| 5. Describes recruitment procedure of validation cohort? | Yes | No | Yes |
| 5a. Inclusion criteria? | Yes | No | Yes |
| 5b. Exclusion criteria? | Yes | No | No |
| 6. Describes patient sampling? (random, consecutive, all, etc.) | Yes | Yes | Yes |
| 7. Describes data collection? | Yes | Yes | Yes |
| 7a. Who identified patients and ensured selection adhered to patient recruitment criteria? | N/A | N/A | N/A |
| 7b. Who collected data? | N/A | N/A | N/A |
| 7c. A priori data collection form? | N/A | N/A | No |
| 7d. How was disease classified? | Yes | Yes | Yes |
| 8. Was there a split sample (i.e. revalidation using a separate cohort) | No | No | No |
| 9. Describe number, training and expertise of persons reading reference standard? | No | No | N/A |
| 10. If >1 person reading reference standard, is kappa quoted? | No | Yes | N/A |
| 11. Were the readers of the reference (validation) test blinded to the results of the classification by administrative data for that patient/ (e.g. Was the reviewer of the charts blinded to how the chart was billed?) | No | No | Yes |
| 12. Describes methods of calculating/comparing diagnostic accuracy? | Yes | Yes | Yes |
| 13. Report when study done, start/end dates of enrollment | Yes | Yes | Yes |
| 14. Describe number of people who satisfied inclusion/exclusion criteria? | Yes | No | Yes |
| 15. Study flow diagram? | No | No | Yes |
| 16. Reports distribution of disease severity? | Yes | No | No |
| 17. Report cross-tabulation of index tests by results of reference standard | Yes | Yes | No |
| 18. Reports at least 4 estimates of diagnostic accuracy? (estimates reported in included studies) | Yes | Yes | Yes |
| 18a. Sensitivity | Yes | Yes | Yes |
| 18b. Specificity | Yes | Yes | Yes |
| 18c. PPV | Yes | Yes | Yes |
| 18d. NPV | Yes | Yes | Yes |
| 18e. Likelihood ratios | No | No | No |
| 18f. Kappa | No | Yes | Yes |
| 18g. Area under the ROC curve/c-statistic | No | No | No |
| 18h. Accuracy/ agreement | No | No | No |
| 18i. Other | No | No | No |
| 19. Was the accuracy reported for any subgroup (e.g. age, geography, different sexes, and so on). | No | No | No |
| 20. If PPV/NPV reported, does ratio of cases/controls of validation cohort approximate prevalence of condition in the population? | No | No | No |
| 21. Reports 95 CIs for each of above? | No | No | Yes |
| 22. Discusses the applicability of the findings? | Yes | Yes | Yes |

*N/A = Not Applicable; PPV = Positive Predictive Value; NPV= Negative Predictive Value; ROC= Receiver-Operator Curve; CI= Confidence Interval
