# Supplementary material for: Systematic review and assessment of validated case definitions for depression in administrative data
Source: BMC Psychiatry. 2014 Oct 17;14:289. doi: 10.1186/s12888-014-0289-5 (PMC4201696; doi:10.1186/s12888-014-0289-5)
Supplement: Additional file 2: — Reasons for Systematic Review Full-Text Article Exclusion. This file presents a table of all excluded full-text articles from the systematic review and the reasons for their exclusion. [file 12888_2014_289_MOESM2_ESM.docx]

Additional File 2. Reasons for Systematic Review Full-Text Article Exclusion

| **Article** | **Reason for Exclusion** |
| --- | --- |
| Bock, 2009 | Did not report diagnostic measures |
| Boudreau, 2004 | Did not validate ICD codes |
| Boydell, 1995 | Did not validate ICD codes |
| Corey-Lisle, 2002 | Did not report diagnostic measures |
| Crawford, 2010 | Did not report diagnostic measures |
| Damush, 2008 | Specific sub-population |
| De Souza, 2010 | Did not validate ICD codes (ICD used as gold-standard) |
| Eguale, 2010 | Did not validate ICD codes |
| Frayne, 2010 | Specific sub-population |
| Gambassi, 1998 | Did not validate ICD codes (ICD used as gold-standard) |
| Gardarsdottir, 2009 | Did not validate ICD codes |
| Greenberg, 2004 | Did not report diagnostic measures |
| Hansen, 2010 | Did not validate ICD codes |
| Hippsley-Cox, 2004 | Did not validate ICD codes |
| Jakobsen, 2008 | Did not use administrative data |
| Kerr, 2000 | Did not validate ICD codes |
| Kim, 2012 | Did not validate depression |
| Kramer, 2003 | Did not report diagnostic measures |
| Maier, 1986 | Did not validate ICD codes (ICD used as gold-standard) |
| McGregor, 2010 | Did not validate ICD codes |
| Niebuhr, 2006 | Did not report diagnostic measures |
| O’Neill, 2008 | Did not validate ICD codes |
| Pavlin, 2004 | Did not validate ICD codes (ICD used as gold-standard) |
| Pederson, 2001 | Did not validate ICD codes |
| Rawson, 1997 | Did not report diagnostic measures |
| Solberg, 2006 | Required pharmacy data |
| Spettell, 2003 | Required pharmacy data |
| Tannenbaum, 2009 | Did not report diagnostic measures |
| Taylor, 2008 | Did not report diagnostic measures |
| Van Os, 2006 | Did not report diagnostic measures |
| van Weel-Baumgarten, 2000 | Did not validate ICD codes |
| Vilalta-French, 2006 | Did not report diagnostic measures |
| West, 2000 | Required pharmacy data |
